# Induction of Multidrug Tolerance in *Plasmodium falciparum* by Extended Artemisinin Pressure

**DOI:** 10.3201/eid2110.150682

**Published:** 2015-10

**Authors:** Sandie Ménard, Tanila Ben Haddou, Arba Pramundita Ramadani, Frédéric Ariey, Xavier Iriart, Johann Beghain, Christiane Bouchier, Benoit Witkowski, Antoine Berry, Odile Mercereau-Puijalon, Françoise Benoit-Vical

**Affiliations:** Université de Toulouse, Toulouse, France (S. Ménard, T. Ben Haddou, A.P. Ramadani, X. Iriart, B. Witkowski, A. Berry, F. Benoit-Vical);; Centre de Physiopathologie de Toulouse-Purpan, Toulouse (S. Ménard. X. Iriart, A. Berry);; Laboratoire de Chimie de Coordination du Centre National de la Recherche Scientifique, Toulouse (T. Ben Haddou, A.P. Ramadani, B. Witkowski, F. Benoit-Vical);; Institut Pasteur, Paris, France (F. Ariey, J. Beghain, C. Bouchier, O. Mercereau-Puijalon);; Centre Hospitalier Universitaire de Toulouse, Toulouse (X. Iriart, A. Berry)

**Keywords:** artemisinin, Plasmodium falciparum, parasites, multidrug tolerance, antimicrobial resistance, drug pressure, atovaquone, quinolone, antifolate, malaria, Southeast Asia

## Abstract

*Plasmodium falciparum* resistance to artemisinin derivatives in Southeast Asia threatens global malaria control strategies. Whether delayed parasite clearance, which exposes larger parasite numbers to artemisinins for longer times, selects higher-grade resistance remains unexplored. We investigated whether long-lasting artemisinin pressure selects a novel multidrug-tolerance profile. Although 50% inhibitory concentrations for 10 antimalarial drugs tested were unchanged, drug-tolerant parasites showed higher recrudescence rates for endoperoxides, quinolones, and an antifolate, including partner drugs of recommended combination therapies, but remained susceptible to atovaquone. Moreover, the age range of intraerythrocytic stages able to resist artemisinin was extended to older ring forms and trophozoites. Multidrug tolerance results from drug-induced quiescence, which enables parasites to survive exposure to unrelated antimalarial drugs that inhibit a variety of metabolic pathways. This novel resistance pattern should be urgently monitored in the field because this pattern is not detected by current assays and represents a major threat to antimalarial drug policy.

## Abstract 2

Tolerance is not detected by current assays and represents a major threat to antimalarial drug policy.

During the past decade, increased commitment and investments in malaria control have markedly reduced malaria-related illness and death in many malaria-endemic areas (*1*). This progress is threatened by emergence of resistance of *Plasmodium falciparum* to artemisinin derivatives used in combination with another drug as first-line therapy for uncomplicated malaria. *P. falciparum* resistance to these derivatives is widespread across Southeast Asia (*1**–**6*) and has been reported in other parts of the world (*7**,**8*). Artemisinin resistance decreases parasite clearance rates and exposes larger numbers of parasites to antimalarial drugs in vivo, but whether it drives selection of higher-grade artemisinin resistance or resistance to the partner drug is unknown. To address this critical question and anticipate potential changes upon prolonged pressure, models are needed. We used the unique in vitro evolution model of F32-ART parasites selected from the African F32-Tanzania clonal line by using multiple dose-escalating artemisinin pressure to study the effect of extended artemisinin pressure on susceptibility to other antimalarial drugs.

We previously reported that artemisinin resistance results from the capacity of young intraerythrocytic F32-ART parasites (ring-stage parasites) to arrest their cell cycle and enter a quiescence state (*9**,**10*). This finding was also observed with artemisinin-resistant parasites from Cambodia (*11**,**12*). Acquisition of a point mutation in the propeller region of the K13 protein after ≈30 drug pressure cycles was on the critical path to artemisinin resistance in the F32-ART lineage (*10*). Genome editing studies confirmed the central role of the K13 locus in artemisinin resistance of the F32 parasites, as well as clinical isolates from Cambodia and diverse laboratory lines (*10**,**13*). A mutant K13 propeller domain has now been associated with artemisinin resistance in Cambodia and in the Greater Mekong Region (*3**–**6**,**14**,**15*). Thus, the F32-ART experimental evolution model proved to be highly relevant in understanding *P. falciparum* artemisinin resistance in the field.

Here, we report the susceptibility profile of F32-ART5 parasites selected after 5 years of escalating artemisinin pressure and assess their susceptibility to this drug and other endoperoxides, as well as unrelated molecules from different chemical classes that inhibit distinct parasite metabolic pathways. We monitored survival and recrudescence after drug exposure, in addition to common monitoring of proliferation. We show that prolonged pressure with only artemisinin results in parasites with a novel pluriresistance phenotype that is highly reminiscent of multidrug tolerance of persister bacteria. Dormancy or stress-induced quiescence is a hallmark of bacterial persistence against a variety of antimicrobial drugs (*16*), and periodic exposure to high doses of bactericidal drugs selects increased levels of persister bacteria (*17**,**18*). An analogous multidrug tolerance/resistance is induced in malaria parasites by extended exposure to high doses of artemisinin, which provides parasites with the capacity to survive lethal doses of diverse classes of antimalarial drugs, including molecules used as drug partners in currently recommended first-line combination therapies.

## Materials and Methods

### Chemicals and Drugs

Chloroquine diphosphate, mefloquine, quinine, and pyrimethamine were obtained from Sigma-Aldrich (Saint-Quentin-Fallavier, France). Artemisinin was obtained from TCI Europe N.V. (Eschborn, Germany) and atovaquone from GlaxoSmithKline (Brentford, UK). Artesunate was obtained from Sanofi (Paris, France) and artemether from Rhone Poulenc Rorer (Paris, France). Dihydroartemisinin and monodesetylamodiaquine (amodiaquine/AQ) were obtained from WWARN (http://www.wwarn.org/). Artemisone was synthesized according to published protocols (*19*).

### Parasites and Parasite Culture

For selection of artemisinin-resistant *P. falciparum* parasites, asynchronous cultures of the F32-Tanzania clone were adjusted to a parasitemia of 5%–7% and grown in the presence of increasing doses of artemisinin (range 10 nmol/L–9 µmol/L) for 24 h for the first 3 years of drug pressure, which resulted in F32-ART3 (*9*). In the next 2 years, each drug-pressure cycle lasted 48 h (drug dose range 9 µmol/L–18µmol/L), which resulted in F32-ART5 (*10*) (Technical Appendix Figure 1). To ensure maintenance of the phenotypic characteristics, parasites were cultivated under regular drug pressure. F32-ART5 parasites studied were collected during the period extending from the 120th to the 123rd artemisinin pressure cycle. Phenotypic and genomic analysis showed no differences in parasites from these 4 (120–123) drug pressure cycles. F32-ART5 and its sibling drug-sensitive F32-TEM, cultured without artemisinin, were cultivated in parallel by using the method of Trager and Jensen with modifications (*9**,**20*).

### Standard Isotopic Drug Susceptibility Assay

The standard isotopic 48-h ^3^H-hypoxanthine-based test (*21*) was used, with minor modifications, to assess the sensitivity of F32-ART5 and F32-TEM lines to 10 antimalarial drugs (*9**,**20*). The 50% inhibitory concentrations (IC_50_s) were determined after 48 h of incubation by using ICEstimator software (http://www.antimalarial-icestimator.net).

### Drug Recrudescence Assay

Synchronized F32-TEM and F32-ART5 ring stages were exposed to drug for 48 h, washed, and placed in drug-free medium. A preliminary drug screening (range 2-fold to >4,000-fold the IC_50_) enabled determination of the most appropriate antimalarial drug dose to discriminate the phenotype response of both lines in the recrudescence assay. Parasitemia was monitored daily to determine the time to recrudesce to the initial parasitemia (*9*). To evaluate artemisinin resistance of older parasite stages, 24-hour-old trophozoites were exposed for 48 h to 3.5 µmol/L artemisinin, and recrudescence was monitored.

### Ring-Stage Survival Assay

For the ring-stage survival assay (RSA), ring-stage parasites (0–3 h postinvasion [RSA^0–3 h^] or 13–16 h postinvasion [RSA^13–16 h^]) from highly synchronous cultures were exposed to 700 nmol/L dihydroartemisinin or 0.1% dimethyl sulfoxide (DMSO) for 6 h, washed, and cultivated for 66 h in standard culture conditions as described (*10**–**12*). Survival rates were calculated after microscopic examination of Giemsa-stained blood smears as the proportion of viable second-generation parasites in wells containing drug compared with that in wells containing DMSO (*11**,**12*). Blinded slides were read by >2 expert microscopists. RSA^0–3 h^ was also performed with 0–3-h postinvasion ring-stage parasites exposed for 6 h to atovaquone (3 µmol/L) or amodiaquine (0.3 µmol/L).

### Ring-Stage Growth Arrest Assay

The ring-stage growth arrest assay (*11*) was used with modifications. In brief, synchronized ring-stage cultures (3%–5% parasitemia, 2.5% hematocrit) were treated with 5% sorbitol to lyse mature stages immediately after a 24-h exposure to 11 µmol/L artemisinin or 0.1% DMSO. Cultures were then resuspended in drug-free culture medium, and parasite counts were monitored microscopically daily until day 28 for sorbitol-treated or non–sorbitol-treated cultures exposed to artemisinins. Time to recrudescence was the time to recrudesce to the initial parasitemia. For the control (DMSO) culture, results were expressed as percentage parasite density 24 h after treatment with sorbitol compared with that for cultures not treated with sorbitol. Results were determined in 5 independent experiments.

### Whole-Genome Sequencing

Whole-genome sequencing of F32-ART5 collected after 123 pressure cycles was performed by using paired-reads sequencing technology (Illumina, Inc., San Diego, CA, USA). Sequences were compared with those of F32-ART5 collected after 120 pressure cycles (*10*), F32-TEM, and reference strain 3D7, as reported (*10*).

### Statistical Analysis

Statistical tests were performed by using SigmaStat version 2.03 (Heame Scientific Software, Chicago, IL, USA). Recrudescence curves (Technical Appendix Figure 2) were analyzed by using the Mantel-Cox test and GraphPad Prism software (GraphPad Inc., San Diego, CA, USA) (*5*). Differences in comparisons were considered significant if p values were <0.05.

## Results

### Effect of Long-term Artemisinin Pressure on IC_50_s for Antimalarial Drugs

F32-ART5 and F32-TEM (cultivated for the same duration in the absence of drug pressure) parasites had similar, low IC_50_s for 4 artemisinin derivatives in the standard isotopic drug susceptibility assay. The same result was observed for quinolines, an antifolate, or atovaquone (Table 1). Moreover, both lines had similar IC_90_s and IC_99_s for all antimalarial drugs tested. Thus, the standard chemosensitivity assay shows absence of proliferation of F32-ART5 and F32-TEM parasites in presence of all 10 antimalarial drugs tested.

**Table 1:** Susceptibility of *Plasmodium falciparum* F32-ART5 and F32-TEM lineages to 10 antimalarial drugs*

Drug	F32-TEM				F32-ART5			p value†
	IC_50_	IC_90_	IC_99_		IC_50_	IC_90_	IC_99_	
Artemisinin	14.2 (13–19.5)	20.9 (16.3–26.4)	29.2 (20.7–39.2)		17.7 (14.2–21.9)	31.2 (23–37.2)	50.7 (37.2–63.3)	0.686
Artesunate	3.9 (2.7–5.1)	6.4 (4.7–7.9)	11.1 (9.4–13.4)		3.8 (3.2–5.6)	6.1 (4.7–7.4)	11.1 (7.7–13.4)	0.886
Artemisone	0.6 (0.3–0.9)	0.7 (0.5–0.9)	0.9 (0.6–1.1)		0.5 (0.3–0.6)	1.2 (1.1–1.3)	3.5 (3.3–3.8)	1.000
Artemether	7.2 (7–7.5)	9 (8.9–9.2)	11.5 (11.2–11.7)		7.2 (6.8–7.5)	8.9 (8.7–9.2)	11.4 (11.1–11.7)	1.000
Chloroquine	32.9 (23.64–45.6)	55.9 (42.9–58.7)	66.2 (52.1–72.1)		28.2 (24.9–33.5)	46.2 (44.7–52.4)	83.4 (76.8–100.5)	0.886
Quinine	105.8 (77.8–136.1)	302.7 (219.9–420.7)	938 (718.5–1,482.5)		118.8 (84.2–156.5)	341.6 (259.7–421.5)	1,026 (742.2–1,439)	0.486
Amodiaquine	25.1 (17.1–33.1)	53.1 (51.7–54.4)	113.4 (88.7–138)		29.6 (28.4–30.9)	51.8 (47.5–56.2)	78.05 (66.5–89.7)	1.000
Mefloquine	68.2 (54.7–88.2)	142.3 (130.9–159.6)	312.9 (224–437)		63.1 (59.4–73.1)	114.1 (101–153.7)	220.9 (161.6–448.5)	0.886
Pyrimethamine	105.0 (90–120.1)	184.6 (167.1–202.1)	385.9 (288–483.8)		74.7 (73.3–76.1)	270.5 (268.7–272.3)	1,102.6 (1,076–1,129)	0.333
Atovaquone	2.8 (2–2.9)	12.7 (10.8–15.8)	95.8 (87.2–104)		1.5 (0.4–3)	7.5 (2.6–12.9)	52.1 (31–64.6)	0.486

*IC_50_, 50% inhibitory concentration; IC_90_, 90% inhibitory concentration; IC_99_, 99% inhibitory concentration. Values were obtained by using the standard isotopic susceptibility assay. Drug concentrations are in nanomoles/liter. Values are medians (25%–75% interquartile ranges). All assays were performed in triplicate. †Data distribution was non-Gaussian. The p values were calculated by using nonparametric Mann-Whitney rank-sum test results for IC_50_s. Similar tests were performed to obtain IC_90_s and IC_99_s, and no differences were observed between F32-ART5 and F32-TEM for any drug tested.

### Survival of F32-ART5 against High-Dose Artemisinin after Drug–Induced Quiescence

In the RSA^0–3h^, in which young ring stages were exposed to 700 nmol/L dihydroartemisinin for 6 h, the survival rate of F32-ART5 parasites was similar to that for F32-ART3, and much higher than survival rates for F32-TEM and F32-ART parasites before acquisition of the K13 M476I mutation (*10*) (Table 2; Technical Appendix Figure 1). Moreover, survival rates of F32-ART5 parasites after 122 and 123 drug pressure cycles were similar.

**Table 2:** RSA values for *Plasmodium falciparum* F32-TEM and F32-ART lineages and recrudescence times for trophozoite parasite stages after a 48-h exposure to artemisinin*

Artemisinin pressure cycle	Dose, µmol/L	RSA^0–3 h^			RSA^13–16 h^			Recrudescence time for trophozoite stage, d	
		Median survival rate† (IQR)	No. assays‡		Survival rate†	No. assays‡		Median (IQR)	No. assays‡
0 (F32-TEM)	0	0 (0–0.03)§	5		0	1		17.5 (17–18)	2
12	0.02	0 (0–0)	2		0	1		ND	NA
17	0.04	0 (0–0.07)¶	3		0	1		ND	NA
48	2.7	11.7 (10.3–14.6)¶	3		2.5	1		ND	NA
115	8.9	6.8 (5.9–15.9)	3		2.1	1		ND	NA
122	9	12.8 (10.6–14.5)¶	3		3.8	1		ND	NA
123	10	9.5 (8.1–11.8)§	4		2.9	1		11 (10.3–12.5)	3

*RSA, ring-stage survival assay; IQR, interquartile range; ND, not determined; NA, not applicable. †Survival rates are expressed as percentage of parasites remaining alive after drug treatment compared with mock-treated culture. ‡Assays that fulfilled criteria for successful culture. §Significant survival rate difference (by Mann Whitney rank sum test, p<0.05) between F32-ART5 and its sibling line F32-TEM. ¶Data were obtained from Ariey et al. (*10*).

**Figure 1:**
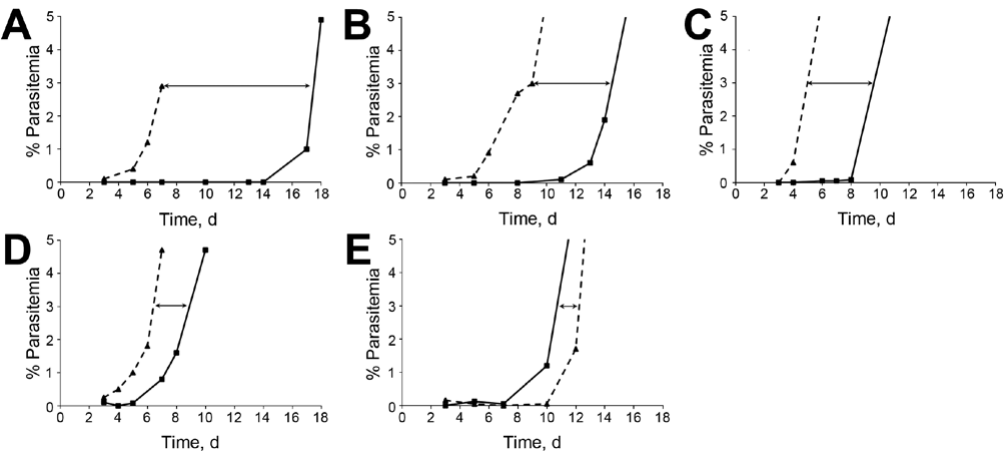
In vitro drug survival assays for *Plasmodium falciparum*. Representative curves for kinetic recrudescence of synchronous ring-stage parasites from F32-ART5 lineage (dashed lines) and F32-TEM lineage (solid lines) parasite cultures after a 48-h exposure to A) 11 μmol/L artemisinin; B) 62 nmol/L amodiaquine; C) 241 nmol/L mefloquine; D) 4 μmol/L pyrimethamine; and E) 7 μmol/L atovaquone. Differences in recrudescence between both parasite lines are indicated by doubled-headed arrows.

**Figure 2:**
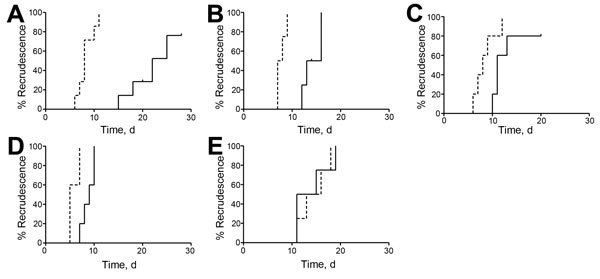
Recrudescence curves of synchronous ring-stage parasites of *Plasmodium falciparum* F32-ART5 (dashed lines) and F32-TEM (solid lines) after a 48-h exposure to A) 11 µmol/L artemisinin; B) 62 nmol/L amodiaquine; C) 241 nmol/L mefloquine; D) 4 µmol/L pyrimethamine; and E) 7 µmol/L atovaquone. Curves show the percentage of parasite recrudescence (i.e., cultures having reached day 0 parasite density) vs. time. A log-rank (Mantel-Cox) test was used for statistical analysis, and corresponding p values are reported in Table 3. Small black vertical tick marks indicate individual F32-TEM lines whose recrudescence times have been right-censored because the parasite line did not recrudesce during the monitoring study.

In the recrudescence assay, in which parasites were exposed to 11 µmol/L artemisinin or 18 µmol/L artemisinin for 48 h, F32-ART5 recrudesced 11 days earlier than F32-TEM (Figure 1, panel A; Figure 2, panel A; Table 3). This finding is consistent with the previously reported recrudescence profile of F32-ART3 parasites (*9*).

**Table 3:** Recrudescence parameters of *Plasmodium falciparum* F32-ART5 and F32-TEM lineages exposed to 10 antimalarial drugs*

Drug	Drug dose	No. experiments†	Median (range) recrudescence time, d‡		Mean ± SEM difference of recrudescence time, d¶	p value‡
			F32-ART5	F32-TEM§		
Artemisinin	11 µmol/L	7	8 (6–11)	22 (15–>28)	11.5 ± 1.5	<0.001
	18 µmol/L	3	7 (6–9)	19 (17–20)	11.3 ± 1.3	0.024
Artesunate	1.3 µmol/L	3	8 (7–10)	16 (15–18)	8 ± 1.5	0.024
	2.6 µmol/L	3	8 (7–10)	16 (15–18)	8 ± 1.5	0.024
Artemisone	1.2 µmol/L	3	8 (7–10)	20 (18–20)	11 ± 1.5	0.024
	2.5 µmol/L	3	8 (7–11)	21 (20–28)	14.3 ± 1.4	0.025
Artemether	1.7 µmol/L	2	7.5 (7–8)	15 (15–15)	7.5 ± 0.5	0.089
	3.4 µmol/L	2	7.5 (7–8)	16 (16–16)	8.5 ± 0.5	0.089
Chloroquine	78 nmol/L	4	10 (7–11)	13 (11–20)	4.8 ± 1.5	0.028
Quinine	43 µmol/L	5	10 (8–14)	13 (10–>20)	2.7 ± 0.9	0.086
Amodiaquine	62 nmol/L	4	7.5 (7–9)	14.5 (12–16)	6 ± 0.6	0.006
Mefloquine	241 nmol/L	5	8 (6–12)	11 (10–>20)	3 ± 1.1	0.044
Pyrimethamine	4 µmol/L	5	5 (5–7)	9 (7–10)	3 ± 0.5	0.008
Atovaquone	3 µmol/L	5	13 (3–27)	12 (3–21)	−1.4 ± 1.3	0.730
	7 µmol/L	4	14.5 (11–18)	13 (11–19)	−0.5 ± 0.6	0.848

*Recrudescence capacity of F32-TEM and F32-ART5, synchronized at ring-form cultures (0–12-h-old parasites), was evaluated after 48 h of drug treatment. After cultures were washed, parasitemia was monitored daily to determine the recrudescence time, defined as the time to reach day 0 parasitemia. †Each experiment was performed for F32-ART and F32-TEM cultivated in parallel in the same conditions (adjusted to the same initial parasitemia and cultivated with the same lot of erythrocytes and same batch of human serum) to generate paired results. The same number of experiments was performed for each parasite lineage and statistically analyzed. ‡A log-rank (Mantel-Cox) test was used for statistical analysis of recrudescence time in days. §If no parasites were observed at the end of the experiment, the culture was classified as showing no recrudescence, and the recrudescence day was noted as >d. Parasite counts were monitored microscopically daily until day 28 except when the batch of blood needed to be changed (in this instance, the experiment was stopped earlier). ¶Differences in recrudescence time as Gaussian data.

To demonstrate that parasite survival against exposure to artemisinin resulted from quiescence involving a proliferation blockade (*11*), sorbitol treatment was performed immediately after a 24-h exposure of synchronized ring-stage cultures to 11 µmol/L artemisinin in the ring-stage growth arrest assay (*11*). Sorbitol selectively lyses erythrocytes infected with trophozoites and mature parasites, but not young stages. Thus, quiescent forms that do not or minimally develop during the 24-h drug exposure are resistant to sorbitol treatment (*9**,**11*). The ring-stage growth arrest assay showed that F32-ART5 parasites exposed to artemisinin recrudesced at the same time whether treated with sorbitol (7.5 d, range 6.5–9 d) or not treated with sorbitol (7.5 d, range 6.5–9.5 d). In contrast, sorbitol induced a severe survival loss in the control culture exposed to DMSO, in which parasite maturation had proceeded unimpaired, such that the mean survival of sorbitol-treated parasites was only 16% (range 6%–33%) of the survival observed in DMSO-control cultures not treated with sorbitol.

### Resistance of Young Ring-Forms and Older Stages of F32-ART Parasites to Artemisinin

We investigated by using RSA the stage-dependent survival capacity of young rings (0–3 h postinvasion) and older rings (13–16 h postinvasion) of the F32-ART lineage at different steps of the selection process (Table 2). We found a parallel increase in survival rates for both stages, although lower rates were observed for older stages. The marked shift of increased survival of young ring-stage parasites previously reported as occurring at approximately pressure cycle 48 coincided with increased survival of older ring-stage parasites, and both rates remained essentially unchanged subsequently.

Drug recrudescence assays showed that F32-ART5 trophozoites recovered more efficiently after a 48 h exposure to artemisinin than F32-TEM trophozoites (11 and 17.5 days, respectively) (Table 2). These data outlined an unsuspected extended age range of stages surviving treatment with artemisinin.

### Selection of Multidrug Tolerance by Long-term Artemisinin Pressure

We reported previously that F32-ART3 showed cross-resistance to artesunate and remained susceptible to chloroquine (i.e., displayed parental-type recrudescence rates after a 48-h exposure to chloroquine in the recrudescence assay) (*9*). Recrudescence assays were performed with F32-ART5 for 4 endoperoxides and a panel of unrelated antimalarial drugs. F32-ART5 showed increased survival rates to high doses of artesunate, artemisone, or artemether, and recrudescence occurred 7.5–14.3 days earlier than for F32-TEM depending on the endoperoxide and concentration (Table 3; Technical Appendix Figure 2). The >7 days earlier recrudescence for F32-ART5 exposed to artemether compared with that for F32-TEM was not significant because of small numbers of paired-sample experiments.

Shorter recrudescence time for F32-ART5 were consistently observed after a 48-h pulse of amodiaquine, mefloquine (Table 3;Figure 1, 2 panels B, C, respectively) and chloroquine (Table 3; Technical Appendix Figure 2). For quinine, we found a trend for increased survival of F32-ART5 compared with that for F32-TEM. Improved survival of F32-ART5 after exposure to quinine was further suggested by 5/5 F32-ART5 cultures having recrudesced compared with 3/5 F32-TEM cultures (Technical Appendix Figure 2). A shorter recrudescence time for F32-ART5 compared with that for F32-TEM was also observed after a 48-h pulse with pyrimethamine (Table 3; Figure 1, panel D; Figure 2, panel D). Therefore, in vitro drug pressure only with artemisinin selects for enhanced survival rates to endoperoxides, quinolines, and an antifolate.

Activity of atovaquone against F32-ART5 parasites was preserved. After a 48-h atovaquone pulse, recrudescence rates for F32-TEM and F32-ART5 were not different (Figure 1, panel E; Figure 2, panel E; Table 3; Technical Appendix Figure 2). Likewise, RSA performed with atovaquone showed no difference between F32-ART5 and F32-TEM (Technical Appendix). The same result was observed for RSA performed with amodiaquine (Technical Appendix).

### Association of Drug Resistance Genes with Multidrug Tolerance

Because phenotypes described were obtained for F32-ART5 parasites collected during the period corresponding to 120–123 pressures cycles, whole-genome sequencing was conducted, and we compared the sequence of F32-ART5_123_ with that of F32-ART5_120_ (*10*). There was no evidence of genotype modification during the 120th–123rd artemisinin pressure cycles (Technical Appendix Figure 1), which is consistent with highly reproducible phenotypes of various F32-ART5 cultures collected during that period. F32-ART5 did not differ from F32-TEM with regard to resistance gene markers, some of which were wild-type (chloroquine resistance transporter, dihydrofolate reductase, and cytochrome B) and others harbored mutations (multidrug resistance 1, ATPase6, Na^+^/H^+^ exchanger 1, dihydropteroate synthase, multidrug resistance 1 [*mdr1*] and *mdr2*) (Technical Appendix Table). There was no evidence for *mdr1* gene amplification in F32-ART5 (and its lineage) and F32-TEM.

## Discussion

F32-ART5 parasites have a novel drug resistance profile that is reminiscent of multidrug tolerance profile of bacterial persister cells (*16**,**22*). Similar to the unchanged inhibitory antimicrobial drug concentrations for bacterial persisters compared with those for their clonal parent strains, IC_50_s, IC_90_s, and IC_99_s of F32-ART5 parasites were similar to those of F32-TEM for 10 antimalarial drugs. Thus, these malaria parasites would be classified as drug susceptible to all 10 drugs in standard proliferation assays. However, high survival rates were observed after exposure to 4 endoperoxides, as well as increased survival rates to lethal doses of pyrimethamine and quinolines (amodiaquine, mefloquine, chloroquine, and quinine). Thus, the in vitro experimental evolution model we used indicates that *P. falciparum* can acquire after sustained artemisinin pressure the capacity to survive exposure to diverse antimalarial drugs. This novel resistance profile differs from classical multidrug resistance because parasites do not multiply in the presence of the drug and do not harbor any mutations conferring resistance to pyrimethamine or quinolines. This profile is not a fully generalized tolerance phenomenon because F32-ART5 is still susceptible to atovaquone.

The capacity of parasites to enter quiescence after exposure to artemisinin and readily resume growth after drug removal is central to resistance to artemisinin (*9**,**11**,**12*) and is similar to persistence of bacteria after exposure to antimicrobial drugs (*17*). Quiescence, also known as dormancy (for definitions, see Technical Appendix), reflects the cell cycle–arrested status and decreased parasite metabolism of parasites exposed to artemisinin (*23*). Resistance to artemisinin is also associated with increased constitutive expression of unfolded protein response pathways, which are believed to mitigate toxicity of artemisinin (*24*). Quiescence of ring-stage F32-ART5 parasites, which did not develop during a 24-h exposure to artemisinin, was shown by the insensitivity of these parasites to treatment with sorbitol and unaltered recrudescence time in the ring-stage growth arrest assay. Because recrudescence rates for other endoperoxides tested were similar to those observed for artemisinin (Table 3), we conclude that F32-ART5 survives the toxicity of endoperoxides by similar cellular mechanisms.

During this study, we determined that not only young rings but also older parasites developmental stages of the F32-ART lineage survived exposure to artemisinin, which denoted an extended range of stages able to enter quiescence and withstand artemisinin toxicity. Relaxed ability of F32-ART5 for cell cycle arrest in response to drug-induced cellular stresses might affect increased recrudescence rates after exposure to quinolines and pyrimethamine, which inhibit metabolically active mature stages and yet are highly active in the standard susceptibility assay that monitors parasite multiplication. Consistent with this idea, mefloquine was shown to induce cell cycle delay (*25*), including that for ring-stage parasites (*26*). Moreover, the reduced metabolic activity of quiescent older forms is predicted to decrease toxicity of antimalarial drugs that inhibit parasite metabolic pathways, such as hemozoin formation (inhibited by quinolines) or tetrahydrofolic acid synthesis (inhibited by pyrimethamine). In contrast, atovaquone, which inhibits parasitic mitochondrial electron transfer and consequently reduces mitochondrial electron membrane potential, remained fully active for F32-ART5 parasites. This finding is consistent with results that showed that parasites retain their basal mitochondrial metabolism during artemisinin-induced quiescence (*23**,**27*), and that addition of atovaquone to ring-stage parasites does not delay parasite maturation (*26*). Unfortunately, atovaquone resistance is readily selected in the field, being detected a few months after deployment in areas of resistance to artemisinin-based combination therapies (ACTs) (*2*). Nevertheless, our data indicate that drug combinations that include atovaquone could be a useful option in decreasing resistance to ACTs in the field.

Despite its extended age range of stages able to enter quiescence, F32-ART3 was fully susceptible to chloroquine in recrudescence assays conducted with the same protocol (*9*). This finding indicates that tolerance to chloroquine was acquired at a later stage of selection (after the 110th pressure cycle). Whether this tolerance was associated with multidrug tolerance or whether acquisition of multidrug tolerance is a multistep process remains to be clarified. To define more precisely the mechanisms, the number of steps, and putative loci involved in multidrug tolerance an analysis of the cryopreserved intermediate time points of the F32-ART lineage would be needed. None of the mutations that occurred after fixation of M476I in the K13 locus (Technical Appendix Figure 1), is analogous with mutations associated with persistence in bacteria or fungi (*16*), and some mutations affect genes of unknown function.

The nonsense mutation in PF3D7_1115700, which encodes falcipain 2a, a cysteine protease involved in hydrolysis of hemoglobin (*28*), is predicted to decrease artemisinin sensitivity of trophozoite stages by impairing artemisinin-induced exacerbation of oxidative stress caused by hemoglobin degradation products (*29*). Absence of this enzyme should also decrease the amount of hemoglobin degradation products essential to activity of quinolines (*30*) and endoperoxides (*19**,**31*). The nonsense falcipain 2a mutation, which is present in F32-ART3, might contribute to reducing sensitivity of F32-ART5 to artemisinins and some quinolines (other than chloroquine), but it is unlikely for pyrimethamine, whose activity is not related to hemoglobin metabolism.

Reduced sensitivity for chloroquine or pyrimethamine, whose target genes (chloroquine resistance transporter gene and dihydrofolate reductase gene, respectively) are wild type in the F32-ART lineage (Technical Appendix Table 1), might result from mutations in other loci affected at late stages of artemisinin pressure or reflect decreased toxicity because of reduced metabolic activity of quiescent parasites. Detailed analysis of the small number of genes mutated in F32-ART5 parasites is needed to gain insights about the cellular alterations underlying multidrug tolerance. In addition, investigation of the field polymorphism of these candidate genes might provide indications about possible ongoing selection processes. It is worth noting that none of these genes was as reported as having signatures of recent selection in Southeast Asia (*6*) or Africa (*10**,**32*). These findings do not preclude that such selection might eventually occur in K13 mutant parasites, including isolates harboring the M476I K13 mutation (*5**,**33*).

The F32-ART model was relevant for understanding cellular and molecular mechanisms of artemisinin resistance in the field (*9**,**10*). The finding that not only young ring forms but also older developmental stages survived exposure to artemisinin differs from observations with artemisinin-resistant field isolates from Cambodia, whose increased survival seems restricted to younger stages, although a trend for increased survival of older ring stages was observed (*12*). Whether this finding reflects different selection processes in the field in Cambodia and during the in vitro model used here is unclear. This finding could also reflect intrinsic differences of genetic backgrounds in which resistance mutations emerge because F32-ART has a genetic background from Africa (*10*).

Genome editing studies have shown that K13 mutations and parasite genetic background influence RSA^0–3 h^ survival rate of young ring stages (*13*). However, survival of older stages has not been studied. Likewise, clearance half-life and RSA^0–3 h^ survival rates of isolates from Cambodia depend on the K13 mutation type (*10**,**14*). Extended monitoring of phenotypes of mutant parasites from Southeast Asia to older developmental stages and characterizing survival and recrudescence phenotype of parasites from Africa harboring a mutant K13 locus (*34*) are urgently needed to provide information on possible selection processes for multidrug tolerance in the field. It is also necessary to further characterize the stage-dependent phenotype of rare field isolates harboring the M476I mutation, including analysis of possible multidrug tolerance phenotype. The recent emergence of resistance to piperaquine, an ACT partner drug, in western Cambodia is particularly worrisome in this context (*35*). However, none of the assays currently used to monitor drug susceptibility of field isolates is able to detect this novel multidrug tolerance phenotype.

The pleiotropic effect of artemisinin pressure reported is a major concern for ACT-based drug policy because sustained pressure on artemisinin-resistant parasites may drive selection of artemisinin resistance in older parasite stages and result in decreased efficacy of the partner drug in the field. Apart from the retained efficacy of atovaquone that suggests possible alternative combination treatments, multidrug tolerance of F32-ART5 to mefloquine, amodiaquine, and pyrimethamine in vitro is a serious concern because of large-scale use of these drugs as partner drugs in ACTs. Specific assays should be urgently implemented to monitor this novel phenotype in the field that otherwise will remain undetected by current in vitro assays or molecular markers.

**Technical Appendix.** Additional information regarding induction of multidrug tolerance in *Plasmodium falciparum* by extended artemisinin pressure.

## References

[1] World Health Organization. World malaria report, 2014. Geneva: The Organization; 2014.

[2] World Health Organization Global Malaria Programme. Status report on artemisinin resistance. WHO/HTM/GMP/20149, 2014. Geneva: The Organization; 2014.

[3] Takala-Harrison S, Jacob CG, Arze C, Cummings MP, Silva JC, Dondorp AM, Independent emergence of *Plasmodium falciparum* artemisinin resistance mutations in Southeast Asia. J Infect Dis. 2015;211:670–9. 10.1093/infdis/jiu491 25180241 PMC4334802

[4] Ashley EA, Dhorda M, Fairhurst RM, Amaratunga C, Lim P, Suon S, Spread of artemisinin resistance in *Plasmodium falciparum* malaria. N Engl J Med. 2014;371:411–23. 10.1056/NEJMoa1314981 25075834 PMC4143591

[5] Tun KM, Imwong M, Lwin KM, Win AA, Hlaing TM, Hlaing T, Spread of artemisinin-resistant *Plasmodium falciparum* in Myanmar: a cross-sectional survey of the K13 molecular marker. Lancet Infect Dis. 2015;15:415–21. 10.1016/S1473-3099(15)70032-0 25704894 PMC4374103

[6] Miotto O, Amato R, Ashley EA, MacInnis B, Almagro-Garcia J, Amaratunga C, Genetic architecture of artemisinin-resistant *Plasmodium falciparum.* Nat Genet. 2015;47:226–34. 25599401 10.1038/ng.3189 PMC4545236

[7] Borrmann S, Sasi P, Mwai L, Bashraheil M, Abdallah A, Muriithi S, Declining responsiveness of *Plasmodium falciparum* infections to artemisinin-based combination treatments on the Kenyan coast. PLoS ONE. 2011;6:e26005. 10.1371/journal.pone.0026005 22102856 PMC3213089

[8] Vreden SG, Jitan JK, Bansie RD, Adhin MR. Evidence of an increased incidence of day 3 parasitaemia in Suriname: an indicator of the emerging resistance of *Plasmodium falciparum* to artemether. Mem Inst Oswaldo Cruz. 2013;108:968–73. 10.1590/0074-0276130167 24402149 PMC4005544

[9] Witkowski B, Lelievre J, Barragan MJ, Laurent V, Su XZ, Berry A, Increased tolerance to artemisinin in *Plasmodium falciparum* is mediated by a quiescence mechanism. Antimicrob Agents Chemother. 2010;54:1872–7. 10.1128/AAC.01636-09 20160056 PMC2863624

[10] Ariey F, Witkowski B, Amaratunga C, Beghain J, Langlois AC, Khim N, A molecular marker of artemisinin resistant *Plasmodium falciparum* malaria. Nature. 2014;505:50–5. 10.1038/nature12876 24352242 PMC5007947

[11] Witkowski B, Khim N, Chim P, Kim S, Ke S, Kloeung N, Reduced artemisinin susceptibility of *Plasmodium falciparum* ring stages in western Cambodia. Antimicrob Agents Chemother. 2013;57:914–23. 10.1128/AAC.01868-12 23208708 PMC3553720

[12] Witkowski B, Amaratunga C, Khim N, Sreng S, Chim P, Kim S, Novel phenotypic assays for the detection of artemisinin-resistant *Plasmodium falciparum* malaria in Cambodia: in-vitro and ex-vivo drug-response studies. Lancet Infect Dis. 2013;13:1043–9. 10.1016/S1473-3099(13)70252-4 24035558 PMC5015432

[13] Straimer J, Gnadig NF, Witkowski B, Amaratunga C, Duru V, Ramadani AP, K13-propeller mutations confer artemisinin resistance in *Plasmodium falciparum* clinical isolates. Science. 2015;347:428–31. 10.1126/science.1260867 25502314 PMC4349400

[14] Amaratunga C, Witkowski B, Dek D, Try V, Khim N, Miotto O, *Plasmodium falciparum* founder populations in western Cambodia have reduced artemisinin sensitivity in vitro. Antimicrob Agents Chemother. 2014;58:4935–7. 10.1128/AAC.03055-14 24867977 PMC4136061

[15] Amaratunga C, Witkowski B, Khim N, Menard D, Fairhurst RM. Artemisinin resistance in *Plasmodium falciparum.* Lancet Infect Dis. 2014;14:449–50. 10.1016/S1473-3099(14)70777-7 24849722 PMC4573664

[16] Lewis K. Persister cells. Annu Rev Microbiol. 2010;64:357–72. 10.1146/annurev.micro.112408.134306 20528688

[17] Wolfson JS, Hooper DC, Mchugh GL, Bozza MA, Swartz MN. Mutants of *Escherichia-coli* K-12 exhibiting reduced killing by both quinolone and beta-lactam antimicrobial agents. Antimicrob Agents Chemother. 1990;34:1938–43. 10.1128/AAC.34.10.1938 1963289 PMC171968

[18] Moyed HS, Bertrand KP. *hipA*, a newly recognized gene of *Escherichia coli* K-12 that affects frequency of persistence after inhibition of murein synthesis. J Bacteriol. 1983;155:768–75 . 6348026 10.1128/jb.155.2.768-775.1983 PMC217749

[19] Witkowski B, Lelievre J, Nicolau-Travers ML, Iriart X, Njomnang Soh P, Bousejra-Elgarah F, Evidence for the contribution of the hemozoin synthesis pathway of the murine *Plasmodium yoelii* to the resistance to artemisinin-related drugs. PLoS ONE. 2012;7:e32620. 10.1371/journal.pone.0032620 22403683 PMC3293827

[20] Benoit-Vical F, Lelievre J, Berry A, Deymier C, Dechy-Cabaret O, Cazelles J, Trioxaquines are new antimalarial agents active on all erythrocytic forms, including gametocytes. Antimicrob Agents Chemother. 2007;51:1463–72. 10.1128/AAC.00967-06 17242150 PMC1855510

[21] Desjardins RE, Canfield CJ, Haynes JD, Chulay JD. Quantitative assessment of antimalarial activity in vitro by a semiautomated microdilution technique. Antimicrob Agents Chemother. 1979;16:710–8. 10.1128/AAC.16.6.710 394674 PMC352941

[22] Dawson CC, Intapa C, Jabra-Rizk MA. “Persisters”: survival at the cellular level. PLoS Pathog. 2011;7:e1002121. 10.1371/journal.ppat.1002121 21829345 PMC3145784

[23] Chen N, LaCrue AN, Teuscher F, Waters NC, Gatton ML, Kyle DE, Fatty acid synthesis and pyruvate metabolism pathways remain active in dihydroartemisinin-induced dormant ring stages of *Plasmodium falciparum.* Antimicrob Agents Chemother. 2014;58:4773–81. 10.1128/AAC.02647-14 24913167 PMC4135995

[24] Mok S, Ashley EA, Ferreira PE, Zhu L, Lin Z, Yeo T, Drug resistance. Population transcriptomics of human malaria parasites reveals the mechanism of artemisinin resistance. Science. 2015;347:431–5. 10.1126/science.1260403 25502316 PMC5642863

[25] Veiga MI, Ferreira PE, Schmidt BA, Ribacke U, Bjorkman A, Tichopad A, Antimalarial exposure delays *Plasmodium falciparum* intra-erythrocytic cycle and drives drug transporter genes expression. PLoS ONE. 2010;5:e12408. 10.1371/journal.pone.0012408 20811640 PMC2928296

[26] Bohórquez EB, Juliano JJ, Kim HS, Meshnick SR. Mefloquine exposure induces cell cycle delay and reveals stage-specific expression of the *pfmdr1* gene. Antimicrob Agents Chemother. 2013;57:833–9. 10.1128/AAC.01006-12 23208721 PMC3553685

[27] Peatey CL, Chavchich M, Chen N, Gresty KJ, Gray KA, Gatton ML, Mitochondrial membrane potential in a small subset of artemisinin-induced dormant *Plasmodium falciparum* parasites in vitro. J Infect Dis. 2015;212:426–34. 10.1093/infdis/jiv048 25635122

[28] Sijwali PS, Rosenthal PJ. Gene disruption confirms a critical role for the cysteine protease falcipain-2 in hemoglobin hydrolysis by *Plasmodium falciparum.* Proc Natl Acad Sci U S A. 2004;101:4384–9. 10.1073/pnas.0307720101 15070727 PMC384756

[29] Klonis N, Crespo-Ortiz MP, Bottova I, Abu-Bakar N, Kenny S, Rosenthal PJ, Artemisinin activity against *Plasmodium falciparum* requires hemoglobin uptake and digestion. Proc Natl Acad Sci U S A. 2011;108:11405–10. 10.1073/pnas.1104063108 21709259 PMC3136263

[30] Foley M, Tilley L. Quinoline antimalarials: mechanisms of action and resistance and prospects for new agents. Pharmacol Ther. 1998;79:55–87. 10.1016/S0163-7258(98)00012-6 9719345

[31] Robert A, Benoit-Vical F, Claparols C, Meunier B. The antimalarial drug artemisinin alkylates heme in infected mice. Proc Natl Acad Sci U S A. 2005;102:13676–80. 10.1073/pnas.0500972102 16155128 PMC1224611

[32] Conrad MD, Bigira V, Kapisi J, Muhindo M, Kamya MR, Havlir DV, Polymorphisms in K13 and falcipain-2 associated with artemisinin resistance are not prevalent in *Plasmodium falciparum* isolated from Ugandan children. PLoS ONE. 2014;9:e105690. 10.1371/journal.pone.0105690 25144768 PMC4140830

[33] Nyunt MH, Hlaing T, Oo HW, Tin-Oo LL, Phway HP, Wang B, Molecular assessment of artemisinin resistance markers, polymorphisms in the K13 propeller, and a multidrug-resistance gene in the eastern and western border areas of Myanmar. Clin Infect Dis. 2015;60:1208–15. 10.1093/cid/ciu1160 25537878

[34] Kamau E, Campino S, Amenga-Etego L, Drury E, Ishengoma D, Johnson K, K13-propeller polymorphisms in *Plasmodium falciparum* parasites from sub-Saharan Africa. J Infect Dis. 2015;211:1352–5 . 25367300 10.1093/infdis/jiu608 PMC4827505

[35] Saunders DL, Vanachayangkul P, Lon C. Dihydroartemisinin-piperaquine failure in Cambodia. N Engl J Med. 2014;371:484–5. 10.1056/NEJMc1403007 25075853

