# Supplementary material for: Induction of Multidrug Tolerance in Plasmodium falciparum by Extended Artemisinin Pressure
Source: Emerg Infect Dis. 2015 Oct;21(10):1733–41. doi: 10.3201/eid2110.150682 (PMC4593447; doi:10.3201/eid2110.150682)
Supplement: Supplementary file 1 — Technical Appendix. Additional information regarding induction of multidrug tolerance in Plasmodium falciparum by extended artemisinin pressure. [file 15-0682-Techapp-s1.pdf]

Article DOI: http://dx.doi.org/10.3201/eid2110.150682

Induction of Multidrug Tolerance in *Plasmodium falciparum* by Extended Artemisinin Pressure

Technical Appendix

We use the term quiescence, rather than dormancy, to refer to the particular cell cycle arrested status resulting from the severe cellular oxidative insult induced by exposure to artemisinin (*1*). Quiescence refers to a metabolically active, nonreplicating cell that is resistant to multiple environmental insults, whereas dormancy is a common (not induced) condition found in many species and describes the propensity of pathogens or seeds to arrest their growth (*2*,*3*). Several metabolic pathways and organelles, including the mitochondrion and the apicoplast, are reported as active in artemisinin-exposed sensitive parasites (*4*).

It appears that all *P. falciparum* strains whatever their genetic background, enter quiescence after exposure to artemisinins, although the rate of recovery differs among susceptible strains, suggesting that artemisinin-induced dormancy is a conserved trait in *Plasmodium falciparum* parasites (*5*,*6*). This baseline propensity, which is also observed after pyrimethamine or mefloquine treatment and results in late recrudescence (*7*,*8*), probably accounts for the late and low-rate recrudescence of the F32-TEM line.

The situation differs markedly in artemisinin-resistant parasites of the F32-ART lineage that have acquired a 1–3 log higher recovery rate/quiescence capacity after exposure to dihydroartemisinin than susceptible strains (*9*,*10*). We reported that F32-ART3 remained quiescent after long exposure (>96 h) to high doses of artemisinin (>70 µmol/L) and quickly recovered after removal of artemisinin (*1*). This finding indicates a remarkably strong capacity of parasites to withstand toxicity of artemisinin, which is consistent with modifications of metabolic composition of artemisinin-resistant parasites documented by recent transcriptome studies that indicate increased expression of unfolded protein response pathways (*11*). Artemisinin-resistant parasites also display a decelerated progression through the first part of the asexual intraerythrocytic development cycle (*11*), which might favor quiescence upon exposure to artemisinin.

Ring-Stage Survival Assay Performed with Atovoquone and Amodiaquine

A ring-stage survival assay was performed with 0–3-h-old ring-stage F32-ART5 and F32-TEM parasites exposed for 6 h to 3 µmol/L amodiaquine or 0.3 µmol/L atovaquone. Similar, increased survival rates were observed for both lines (27% and 32% for amodiaquine, and 20% and 27% for atovaquone). These results are consistent with the insensitivity of ring stages to quinolines that inhibit mature stages, are inactive on young rings, and require a long incubation time (*12*), and with mode of action of atovaquone that targets mature stages (*13*).

The recently developed ring-stage survival assay monitors susceptibility to fast-acting molecules, such as artemisinin derivatives, and is sensitive for young developmental stages. Therefore, this assay is irrelevant for long-acting molecules and those molecules that exert their inhibitory power at later developmental stages (such as amodiaquine and atovaquone).

Specific assays should be implemented to detect multidrug tolerance in the field. Improvements to the recrudescence assays used are warranted because they are time-consuming and labor-intensive. These assays should be conducted in addition to those currently used in field-based settings. The novel multidrug resistance phenotype should be evaluated for possible emergence in the field alongside possible selection of genuine resistance to the partner drug.

References

1. Witkowski B, Lelievre J, Barragan MJ, Laurent V, Su XZ, Berry A, et al. Increased tolerance to artemisinin in *Plasmodium falciparum* is mediated by a quiescence mechanism. Antimicrob Agents Chemother. 2010;54:1872–7. PubMed http://dx.doi.org/10.1128/AAC.01636-09

2. Rittershaus ES, Baek SH, Sassetti CM. The normalcy of dormancy: common themes in microbial quiescence. Cell Host Microbe. 2013;13:643–51. PubMed http://dx.doi.org/10.1016/j.chom.2013.05.012

3. Geneve RL. Seed dormancy in commercial vegetable and flower species. In: Seed Technology. Washington (DC): Association of Official Seed Analysts; 1998. p. 236–50.

4. Chen N, LaCrue AN, Teuscher F, Waters NC, Gatton ML, Kyle DE, et al. Fatty acid synthesis and pyruvate metabolism pathways remain active in dihydroartemisinin-induced dormant ring stages of *Plasmodium falciparum*. Antimicrob Agents Chemother. 2014;58:4773–81. PubMed http://dx.doi.org/10.1128/AAC.02647-14

5. Cheng Q, Kyle DE, Gatton ML. Artemisinin resistance in *Plasmodium falciparum*: a process linked to dormancy? Int J Parasitol Drugs Drug Resist. 2012;2:249–55. PubMed

6. Teuscher F, Gatton ML, Chen N, Peters J, Kyle DE, Cheng Q. Artemisinin-induced dormancy in *Plasmodium falciparum*: duration, recovery rates, and implications in treatment failure. J Infect Dis. 2010;202:1362–8. PubMed http://dx.doi.org/10.1086/656476

7. Nakazawa S, Kanbara H, Aikawa M. *Plasmodium falciparum*: recrudescence of parasites in culture. Exp Parasitol. 1995;81:556–63. PubMed http://dx.doi.org/10.1006/expr.1995.1149

8. Nakazawa S, Maoka T, Uemura H, Ito Y, Kanbara H. Malaria parasites giving rise to recrudescence in vitro. Antimicrob Agents Chemother. 2002;46:958–65. PubMed http://dx.doi.org/10.1128/AAC.46.4.958-965.2002

9. Ariey F, Witkowski B, Amaratunga C, Beghain J, Langlois AC, Khim N, et al. A molecular marker of artemisinin resistant *Plasmodium falciparum* malaria. Nature. 2014;505:50–5. PubMed http://dx.doi.org/10.1038/nature12876

10. Straimer J, Gnadig NF, Witkowski B, Amaratunga C, Duru V, Ramadani AP, et al. K13-propeller mutations confer artemisinin resistance in *Plasmodium falciparum* clinical isolates. Science. 2015;347:428–31. PubMed http://dx.doi.org/10.1126/science.1260867

11. Mok S, Ashley EA, Ferreira PE, Zhu L, Lin Z, Yeo T, et al. Drug resistance. Population transcriptomics of human malaria parasites reveals the mechanism of artemisinin resistance. Science. 2015;347:431–5. PubMed http://dx.doi.org/10.1126/science.1260403

12. Krishna S, White NJ. Pharmacokinetics of quinine, chloroquine and amodiaquine. Clinical implications. Clin Pharmacokinet. 1996;30:263–99. PubMed http://dx.doi.org/10.2165/00003088-199630040-00002

13. Beerahee M. Clinical pharmacology of atovaquone and proguanil hydrochloride. J Travel Med. 1999;6:S13–7. PubMed

Technical Appendix Table. Genotypic characteristics of *Plasmodium falciparum* F32-ART5 and F32-TEM lineages for known or candidate drug-resistance genes*

Gene	Name	Nucleotide	Amino acid
PF3D7_0709000	*crt*	3D7 type	3D7 type
PF3D7_0523000	*mdr1*	A551T T1512A G1948A A1954G	Y184F N504K D650N N652D
PF3D7_1303500	*nhe1*	C21G C2605T G4140T T4670C	H869Y K1380N F1557S
PF3D7_0417200	*dhfr*	3D7 type	3D7 type
PF3D7_0810800	*dhps*	G426A T1306G	S436A
PF3D7_1447900	*mdr2*	G623A T1268A A1474G A2127C	S208N F423Y I492V I709I
M76611, mal_mito_3	*cytb*	3D7 type	3D7 type

*Shown are sequence characteristics of F32-ART5 parasites surviving pressure cycle 123. Sequence is identical to that of F32-ART5 collected at pressure cycle 120 and to F32-TEM (*9*). Reference sequence is 3D7 (plasmodb.org). *crt*, chloroquine resistance transporter; *mdr1*, multidrug resistance 1; *nhe1*, Na^+/H^+ exchanger 1; *dhfr*, dihydrofolate reductase; *dhps*, dihydropteroate synthase; *cytb*, cytochrome b.

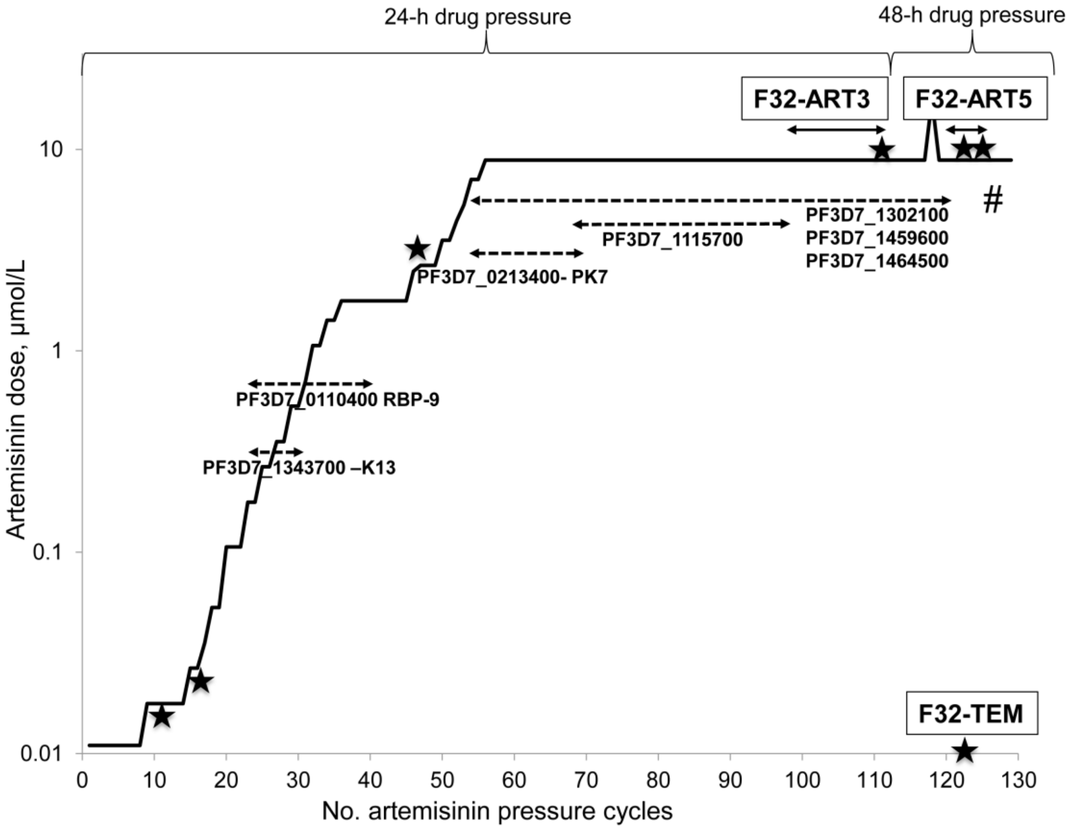

Technical Appendix Figure 1. Stepwise artemisinin selection of *Plasmodium falciparum* F32-ART lineage and sampling for molecular and phenotypic studies. F32-Tanzania parasites were exposed to increasing artemisinin concentrations (*1,2*) for 123 consecutive cycles and analyzed by using whole-genome sequencing and PCR to detect more precise time windows of acquisition of specific mutations. Dashed double-headed arrows indicate selection window during which individual mutations arose and were fixed. The last whole-genome sequencing (#) performed on the F32-ART5 line (drug pressure cycle 123) confirmed the presence of the 8 mutations detected in F32-ART5 from pressure #120 and showed no additional mutation or gene copy amplification. The D56V mutation of PF3D7_0110400 (RBP-9) was acquired between cycles 23 and 39; the M476I mutation of PF3D7_1343700 (K13 locus) was acquired between cycles 23 and 30; the double mutation of codon104, E104stop of PF3D7_0213400 (PK7) was acquired between cycles 56 and 68; the S69stop mutation of PF3D7_1115700 (falcipain2a) was acquired between cycles 68 and 98; the P201T mutation of PF3D7_1302100 (Pfg27) was acquired between cycles 56 and 120; the S292T mutation of PF3D7_1459600 (unknown function) was acquired between cycles 56 and 120; and the N1629S mutation of PF3D7_1464500 (unknown function) was acquired between cycles 56 and 120 (*1*). As *in vitro* phenotyping experiments conducted on F32-ART lineage lasted several months, parasites were cultivated under regular drug pressure to ensure maintenance of the phenotypic characteristics. However, assays were performed once a stable concentration/duration of pressure was achieved. The windows corresponding to F32-ART3 and F32-ART5 are indicated by the solid double-headed arrows. Black stars indicate intermediate lines from the F32-ART lineage tested by using ring-stage survival assay (RSA)$^{0–3\ h}$ and RSA$^{13–16\ h}$ (Table 2).

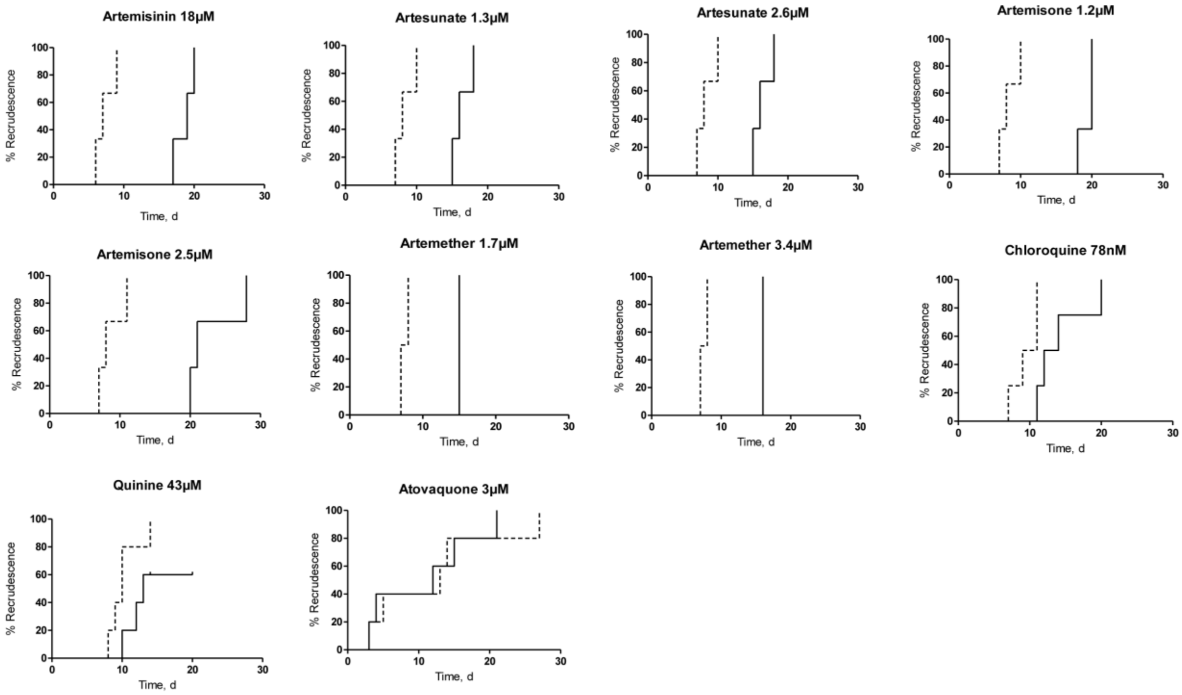

Technical Appendix Figure 2. Recrudescence curves of synchronous ring-stage parasites of *Plasmodium falciparum* F32-ART5 (dashed lines) and F32-TEM (solid lines) after a 48-h exposure to various antimalarial drugs. Curves show the percentage of parasite recrudescence (i.e., cultures having reached day 0 parasite density) vs. time. A log-rank (Mantel-Cox) test was used for statistical analysis, and corresponding *p* values are reported in Table 3. Small black vertical tick marks indicate individual F32-TEM lines whose recrudescence times have been right-censored because the parasite line did not recrudesce during the monitoring study.
